# Supplementary material for: Comparison of Disk Diffusion, E-Test, and Broth Microdilution Methods for Testing In Vitro Activity of Cefiderocol in Acinetobacter baumannii
Source: Antibiotics (Basel). 2023 Jul 20;12(7):1212. doi: 10.3390/antibiotics12071212 (PMC10376138; doi:10.3390/antibiotics12071212)
Supplement: Supplementary file 1 [file antibiotics-12-01212-s001.zip › antibiotics-2487975-supplementary.pdf]

Tables

Table S1. Features of the isolates used in this study.

Study Nr.	ST	Plasmid-borne carbapenemase(s)	Plasmid-borne ESBL	Plasmid-borne additional β-lactamase(s)	ADC	intrinsic oxacillinase	Plasmid-borne 16S RMTase	Cefiderocol MIC	Acquired carbapenem resistance marker(s)
1	2	blaOXA-23		blaTEM-12	blaADC-73	blaOXA-66	*armA*	1	OXA-23
2	2	blaOXA-23		blaTEM-12	blaADC-73	blaOXA-66	*armA*	0.5	OXA-23
3	2	blaOXA-23		blaTEM-12	blaADC-73	blaOXA-66	*armA*	1	OXA-23
4	2	blaOXA-23		blaTEM-12	blaADC-73	blaOXA-66	*armA*	1	OXA-23
5	2	blaOXA-23		blaTEM-12	blaADC-73	blaOXA-66	*armA*	1	OXA-23
6	n.d.	blaOXA-23			blaADC-73	blaOXA-66	*armA*	1	OXA-23
7	604	blaOXA-23		blaTEM-12	blaADC-73	blaOXA-66	*armA*	1	OXA-23
8	n.d.	blaOXA-23			blaADC-175	blaOXA-69		1	OXA-23
9	n.d.	blaOXA-72			blaADC-74	blaOXA-69		64	OXA-72
10	1	blaOXA-72		blaTEM-12	blaADC-81	blaOXA-92		1	OXA-72
11	636	blaOXA-72		blaTEM-12	blaADC-73	blaOXA-66		1	OXA-72
12	2	blaOXA-72			blaADC-30	blaOXA-66		0.5	OXA-72
13	1	blaOXA-72		blaTEM-12	blaADC-81	blaOXA-92		1	OXA-72
14	2	blaOXA-23			blaADC-33	blaOXA-115		2	OXA-23
15	2	-		blaTEM-12	blaADC-30	blaOXA-66		1	-
16	1	blaOXA-23	blaGES-22		blaADC-38	blaOXA-69		1	OXA-23/GES-ESBL
17	2	blaOXA-23			blaADC-30	blaOXA-66		1	OXA-23
18	25	blaNDM-1			blaADC-26	blaOXA-64		4	NDM-1
19	103	blaNDM-2			blaADC-203	blaOXA-70		2	NDM-2
20	1	blaNDM-1/blaOXA-23		blaTEM-12	blaADC-11	blaOXA-69		2	NDM-1/OXA-23
21	107	blaOXA-40			blaADC-87	blaOXA-51		0.5	OXA-40
22	1	blaOXA-23	blaPER-1		blaADC-11	blaOXA-69		8	OXA-23/PER-ESBL
23	20	blaOXA-23		blaTEM-12	blaADC-74	blaOXA-69		0.25	OXA-23
24	1	blaOXA-23			blaADC-187	blaOXA-69		1	OXA-23
25	1	blaOXA-23	blaPER-1		blaADC-11	blaOXA-69		64	OXA-23/PER-ESBL
26	108	-			blaADC-152	blaOXA-132		0.5	-
27	25	blaOXA-58			blaADC-26	blaOXA-64		0.125	OXA-58
28	113	-	blaGES-11		blaADC-57	blaOXA-64		1	GES-ESBL

29	113	blaGES-14			blaADC-57	blaOXA-64		0.5	GES-carba
30	47	blaOXA-58			blaADC-1	blaOXA-260		8	OXA-58
31	3	-			blaADC-119	blaOXA-71		0.5	-
32	15	blaOXA-58			blaADC-2	blaOXA-51		0.25	OXA-58
33	2	-			blaADC-30	blaOXA-109		0.5	-
34	636	blaOXA-72			blaADC-74	blaOXA-66	*armA*	4	OXA-72
35	78	blaOXA-72	blaCTX-M-115	blaCARB-14	blaADC-152	blaOXA-90		0.25	OXA-72/CTX-M-ESBL
36	2	blaOXA-23		blaTEM-12	blaADC-73	blaOXA-66	*armA*	0.5	OXA-23
112	85	blaNDM-1			blaADC-25	blaOXA-94		4	NDM-1
38	25	blaOXA-23			blaADC-26	blaOXA-64	*armA*	0.5	OXA-23
39	2	blaOXA-23			blaADC-73	blaOXA-66	*armA*	1	OXA-23
40	2	blaOXA-23			blaADC-73	blaOXA-66	*armA*	0.5	OXA-23
41	25	blaOXA-23			blaADC-26	blaOXA-64	*armA*	0.25	OXA-23
42	400	-	blaGES-11		blaADC-179	blaOXA-100		1	GES-ESBL
43	2	blaOXA-23			blaADC-73	blaOXA-66	*armA*	0.5	OXA-23
44	492	blaNDM-1/blaOXA-72			blaADC-30	blaOXA-66	*armA*	16	NDM/OXA-72
45	45	blaOXA-72	blaPER-1		blaADC-11	blaOXA-66		64	OXA-72/PER-ESBL
46	2	blaNDM-1/blaOXA-23			blaADC-11	blaOXA-66	*armA*	16	NDM-1/OXA-23
47	2	blaOXA-23		blaTEM-12	blaADC-188	blaOXA-66	*armA*	2	OXA-23
48	2	blaOXA-23		blaTEM-12	blaADC-73	blaOXA-66	*armA*	0.5	OXA-23
49	78	blaOXA-72	blaCTX-M-115	blaCARB-14	blaADC-152	blaOXA-90		0.25	OXA-72/CTX-M-ESBL
50	164	blaOXA-23		blaCARB-16	blaADC-52/199	blaOXA-92		1	OXA-23
51	novel ST	blaOXA-72			blaADC-185	blaOXA-66		1	OXA-72
52	85	blaNDM-1			blaADC-25	blaOXA-94		64	NDM-1
53	164	blaOXA-23/blaOXA-58		blaCARB-16	blaADC-25	blaOXA-91		2	OXA-23/OXA-58
54	2	blaOXA-23		blaTEM-12	blaADC-73	blaOXA-66	*armA*	0.5	OXA-23
55	78	blaOXA-72			blaADC-152	blaOXA-90	*armA*	0.125	OXA-72
56	25	blaOXA-23	blaPER-7		blaADC-26	blaOXA-64	*armA*	64	OXA-23/PER-ESBL
57	575	blaOXA-23	blaPER-7		blaADC-76	blaOXA-144	*armA*	64	OXA-23/PER-ESBL
58	2	blaOXA-23			blaADC-73	blaOXA-66	*armA*	0.5	OXA-23
59	2	blaOXA-23			blaADC-73	blaOXA-66		0.5	OXA-23
60	2	blaOXA-23		blaTEM-12	blaADC-73	blaOXA-66	*armA*	0.5	OXA-23

61	2	blaOXA-23			blaADC-33	blaOXA-115		4	OXA-23
62	2	blaOXA-23		blaTEM-12	blaADC-185	blaOXA-66	*armA*	2	OXA-23
63	2	blaOXA-23		blaTEM-12	blaADC-73	blaOXA-66	*armA*	0.5	OXA-23
64	2	blaOXA-23			blaADC-73	blaOXA-66	*armA*	0.5	OXA-23
65	2	blaOXA-23			blaADC-73	blaOXA-66	*armA*	1	OXA-23
66	592	blaOXA-72	blaCTX-M-115		blaADC-185	blaOXA-69	*armA*	1	OXA-72/CTX-M-ESBL
67	164	blaOXA-23		blaCARB-16	blaADC-25	blaOXA-91		4	OXA-23
68	2	blaOXA-23			blaADC-73	blaOXA-66	*armA*	0.5	OXA-23
69	1902	blaOXA-23	blaPER-7		blaADC-26	blaOXA-64	*armA*	64	OXA-23/PER-ESBL
70	2	blaOXA-23			blaADC-73	blaOXA-66	*armA*	0.5	OXA-23
71	2	blaOXA-23			blaADC-73	blaOXA-66	*armA*	0.5	OXA-23
72	2	blaOXA-23			blaADC-73	blaOXA-66	*armA*	0.5	OXA-23
73	2	blaOXA-23			blaADC-73	blaOXA-66	*armA*	16	OXA-23
74	2	blaOXA-23			blaADC-30	blaOXA-66		1	OXA-23
75	2	blaOXA-23			blaADC-73	blaOXA-66	*armA*	0.5	OXA-23
76	2	blaOXA-23			blaADC-73	blaOXA-66	*armA*	1	OXA-23
77	641	blaOXA-23			blaADC-30	blaOXA-66	*armA*	2	OXA-23
78	25	blaOXA-23	blaPER-7		blaADC-26	blaOXA-64	*armA*	64	OXA-23/PER-ESBL
79	2	blaOXA-23			blaADC-73	blaOXA-66	*armA*	1	OXA-23
80	492	blaNDM-1/blaOXA-72			blaADC-30	blaOXA-66	*armA*	4	NDM-1/OXA-72
81	78	blaOXA-72	CTX-M-115	blaCARB-14	blaADC-152	blaOXA-90	*armA*	1	OXA-72/CTX-M-ESBL
82	641	blaOXA-23			blaADC-30	blaOXA-66	*armA*	4	OXA-23
83	2	blaOXA-23			blaADC-73	blaOXA-66	*armA*	1	OXA-23
84	2	blaOXA-23			blaADC-73	blaOXA-66		1	OXA-23
85	2	blaOXA-23		blaTEM-12	blaADC-25	blaOXA-115	*armA*	8	OXA-23
86	2	blaOXA-23			blaADC-73	blaOXA-66	*armA*	0.5	OXA-23
87	2	blaOXA-23			blaADC-73	blaOXA-66	*armA*	0.5	OXA-23
88	2	blaOXA-23			blaADC-73	blaOXA-66	*armA*	0.5	OXA-23
89	n.d.	blaOXA-72			blaADC-185	blaOXA-66	*armA*	1	OXA-72
90	164	blaOXA-23		blaCARB-16	blaADC-199	blaOXA-91		8	OXA-23
91	2	blaOXA-23/blaOXA-72			blaADC-73	blaOXA-66	*armA*	2	OXA-23/OXA-72
92	2	blaOXA-23/blaOXA-72			blaADC-56	blaOXA-66	*armA*	64	OXA-23/OXA-72
93	2	blaOXA-23			blaADC-73	blaOXA-66	*armA*	0.25	OXA-23

94	492	blaNDM-1/blaOXA-72			blaADC-30	blaOXA-66	*armA*	2	NDM-1/OXA-72
95	164	blaOXA-23		blaCARB-16	blaADC-152	blaOXA-91		8	OXA-23
96	2	blaOXA-23			blaADC-73	blaOXA-66	*armA*	0.5	OXA-23
97	2	blaOXA-23			blaADC-73	blaOXA-66	*armA*	0.5	OXA-23
98	2	blaOXA-23			blaADC-73	blaOXA-66	*armA*	0.5	OXA-23
99	2	blaOXA-23			blaADC-73	blaOXA-66	*armA*	0.5	OXA-23
100	2	blaOXA-23			blaADC-73	blaOXA-66	*armA*	1	OXA-23

ST, sequency type; ADC, *Acinetobacter*-derived cephalosporinase; 16S RMTase, 16S rRNA methyltransferase; MIC, minimal inhibitory concentration

Table S2. **MIC-based susceptibility rates of the *A.baumannii* isolates towards second-line and last-resort antibiotics**

**Antibiotic**	**MIC range (µg/ml)**	**MIC_{50} (µg/ml)**	**MIC_{90} (µg/ml)**	**Breakpoint**				**Susceptible (%)**	**Intermediate (%)**	**Resistant (%)**
				**Source[1, 2]**	**S ≤**	**I =**	**R >**			
**Cefiderocol**	0.125 to >64	1	4	EUCAST PK-PD (*A. baumannii*)	2		2	76 (76)		24 (24)
				CLSI (*A. baumannii*)	4	8	8	83 (83)	5 (5)	12 (12)
**Tigecycline**	0.19-32	2	4	EUCAST (*E. coli, C. koseri*)	0.5		0.5	9 (9)		91 (91)
**Eravacycline**	0.032-4	0.5	1	EUCAST (*E. coli*)	0.5		0.5	61 (61)		39 (39)
**Ceftazidime-avibactam**	1 to >256	>256	>256	EUCAST (*P. aeruginos*a)	8		8	3 (3)		97 (97)
**Ceftolozane-tazobactam**	2 to >256	96	>256	EUCAST (*P. aeruginos*a)	4		4	2 (2)		98 (98)
**Ampicillin-sulbactam**	8 to >256	>256	>256	CLSI (*A. baumannii*)	8/4	16/8	16/8	1 (1)	5 (5)	94 (94)
**Colistin**	0.125-128	0.5	1	EUCAST (*A. baumannii*)	2		2	96 (96)		4 (4)

Table S3. DD-based susceptibility rates of the *A.baumannii* isolates towards first-line antibiotics

Antibiotic	Breakpoint (mm)			Susceptible (%)	Intermediate (%)	Resistant (%)
	S ≥	R <	Source[1, 2]			
**Piperacillin-tazobactam**	21	18	CLSI			100 (100)
**Ceftazidime**	18	15	CLSI		1 (1)	99 (99)
**Cefepime**	18	15	CLSI	1 (1)	2 (2)	97 (97)
**Imipenem**	24	21	EUCAST	1 (1)	2 (2)	97 (97)
**Meropenem**	21	15	EUCAST		5 (5)	95 (95)
**Ciprofloxacin**	50	21	EUCAST			100 (100)
**Levofloxacin**	23	20	EUCAST	1 (1)	2 (2)	97 (97)
**Gentamicin**	17	17	EUCAST	6 (6)		94 (94)
**Tobramycin**	17	17	EUCAST	19 (19)		81 (81)
**Amikacin**	19	19	EUCAST	6 (6)		94 (94)

Figures

Figure S1. Distribution of growth inhibition zones of first-line antibiotics. The vertical dashed and continous lines denote the EUCAST and when not available the CLSI susceptible and resistant CBPs for *A. baumannii*.

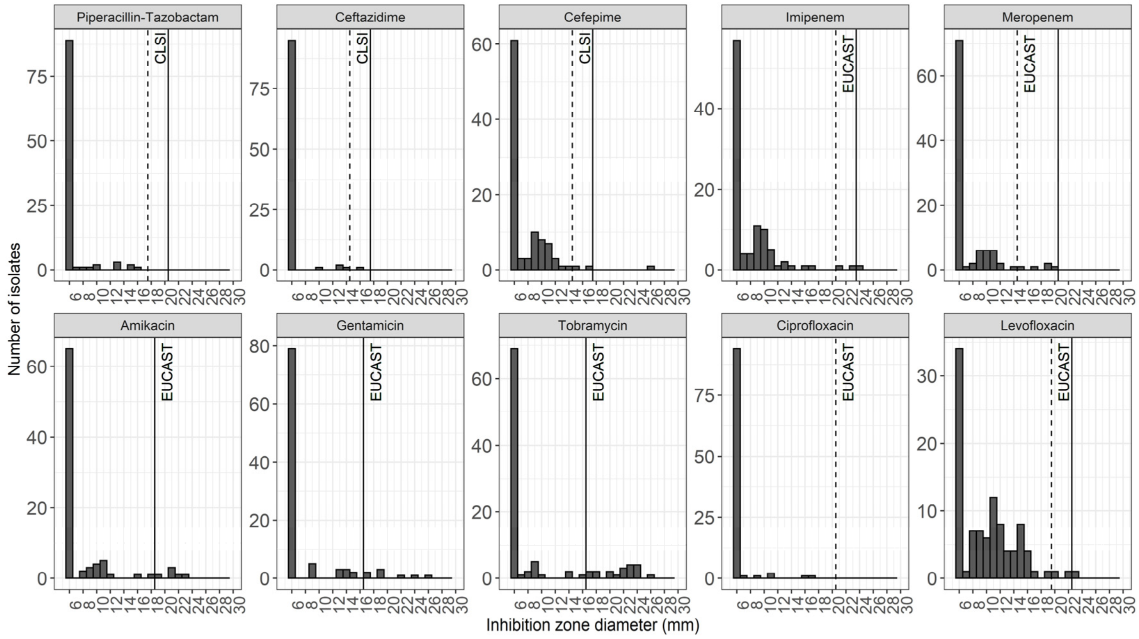

Figure S2. Distribution of MICs of second-line and last-resort antibiotics. The vertical dashed and continous lines denote the EUCAST susceptible and resistant CBPs for *A. baumannii* and when not available for *P. aeruginosa* and *E. coli*. Colistin MICs were determined by BMD, while for the others by E-test.

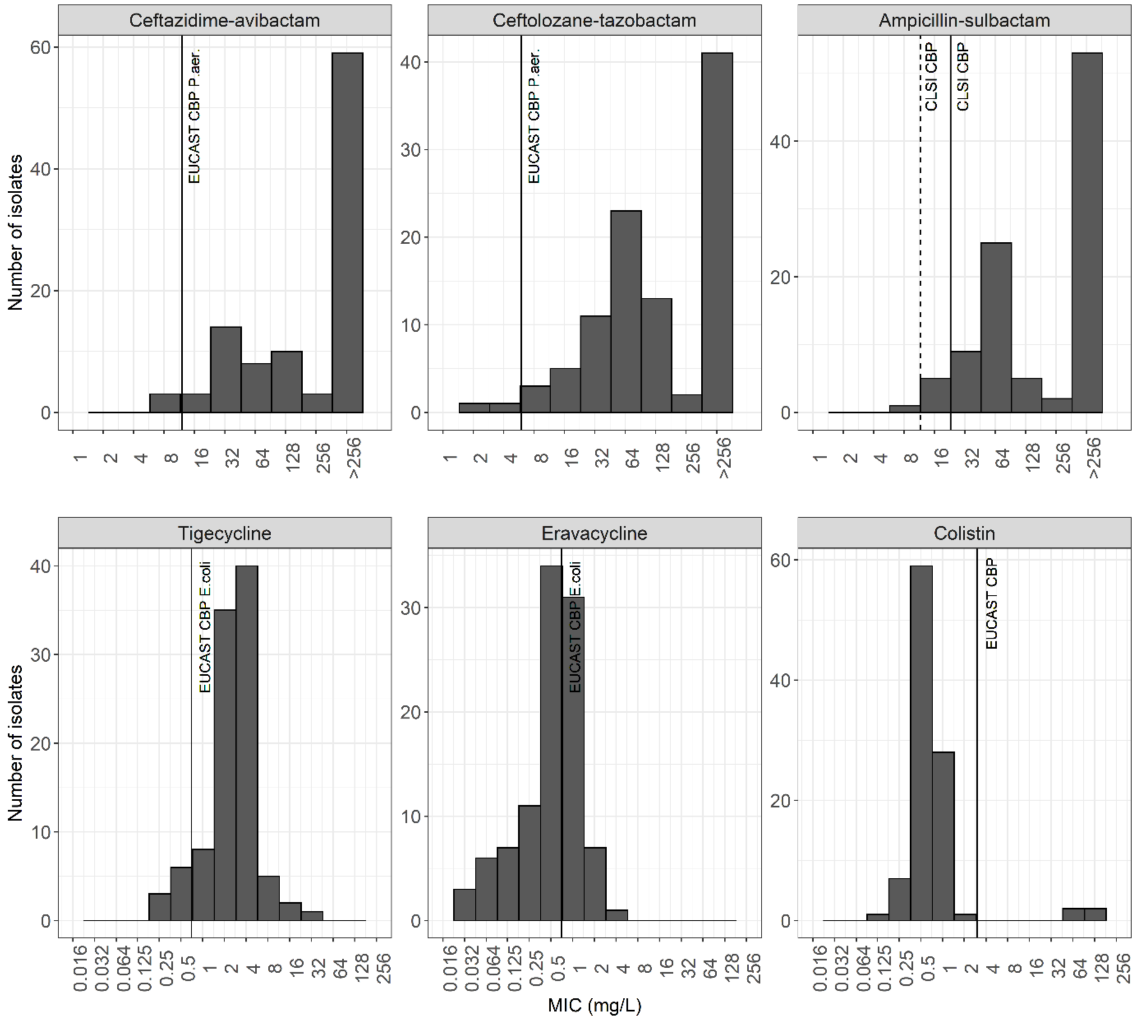

Figure S3. Quality control. Cefiderocol MICs values obtained with the standard BMD, ComASP, UMIC, and by E-test on iron-depleted MH-agar (ID-MH), BioMérieux MH-agar (Biomerieux-MH), Liofilchem MH-agar (Liofilchem-MH) plates, and disc diffusion (DD) growth inhibition zones on ID-MH-agar, Biomerieux-MH-agar and Liofilchem MH-agar plates of *A.baumannii* NCTC13304 and *P.aeruginosa* ATCC27853. Replica numbers are on the x axis and MICs on the y axis. Green and yellow areas reflect the EUCAST targets and ranges for *P.eruginosa* ATCC27853, respectively.

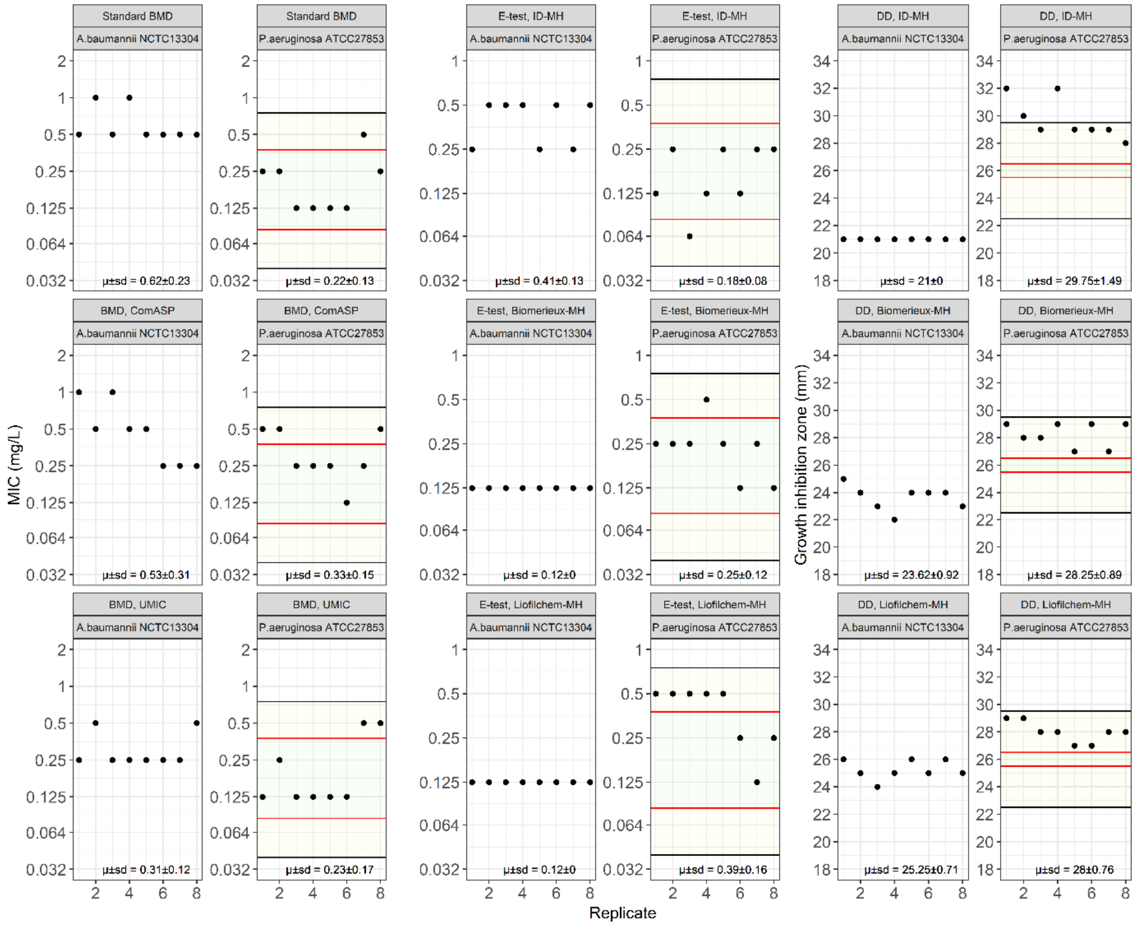

Figure S4. Phylogenetic neighbor joining tree generated in Ridom Seqspere+ based on core genes with associated metadata. In columns from left to right: ST Pasteur, acquired carbapenemase resistance markers, cefiderocol MIC.

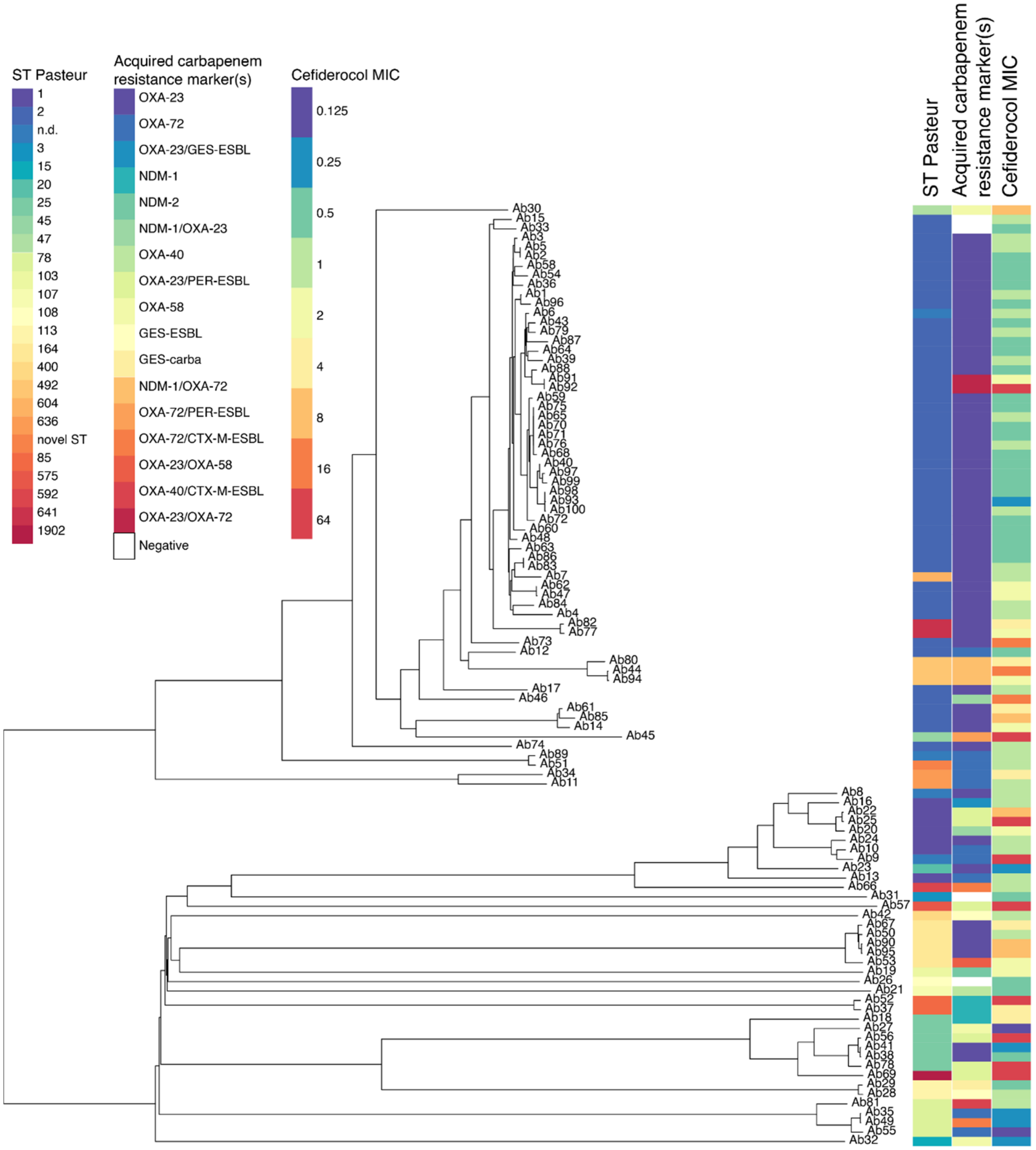

Figure S5. Performance of E-test on different MH-agars and ComASP and UMIC BMD assays. Blant-Altmann comparison between MICs determined by E-test on iron-depleted MH-agar (ID-MH), BioMérieux MH-agar (Biomeriux-MH), Liofilchem MH-agar (Liofilchem-MH) and with the commercial BMD assays ComASP and UMICs versus MICs determined with the standard BMD method. Standard BMD MICs are on the x axis and E-test/ComASP/UMIC MICs on the y axis. Isolates were categorized based on the BMD MICs and the CLSI CBPs for *A. baumannii* (top figures) or the EUCAST PK-PD breakpoint (bottom figures). Standard MICs/E-test or ComASP/UMIC MICs were classified as categorical agreement in green, very major error in red, major error in orange, and minor error in blue. The darkgreen horizontal lines indicate identity between the two methods. The black dashed horizontal lines and numbers indicate the mean $\log_2$ differences of the susceptible (S), intermediate (I) and resistant (R) populations. Percentiles 2.5 and 97.5 are indicated by black dotted horizontal lines. The vertical lines indicate the CLSI CBPs (top) and the EUCAST PK-PD breakpoint (bottom). The gray highlighted areas denote essential agreement (MIC ± 1-fold dilution of the reference MIC).

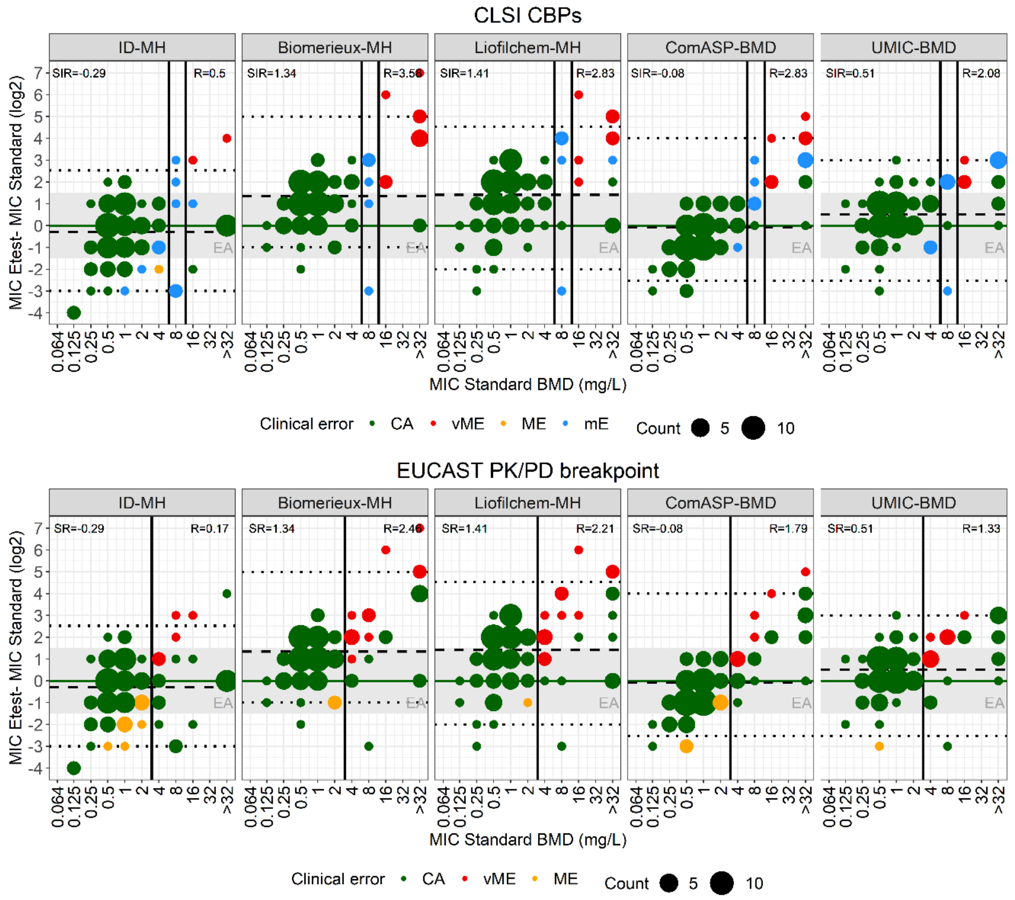

Figure S6. Cefiderocol resistant *A. baumannii* isolate showing colonies emerging within the inhibition zones of DD and E-test performed on iron-depleted MH-agar (ID-MH), but not on BioMérieux MH-agar (Biomerieux-MH) and Liofilchem MH-agar (Liofilchem-MH).

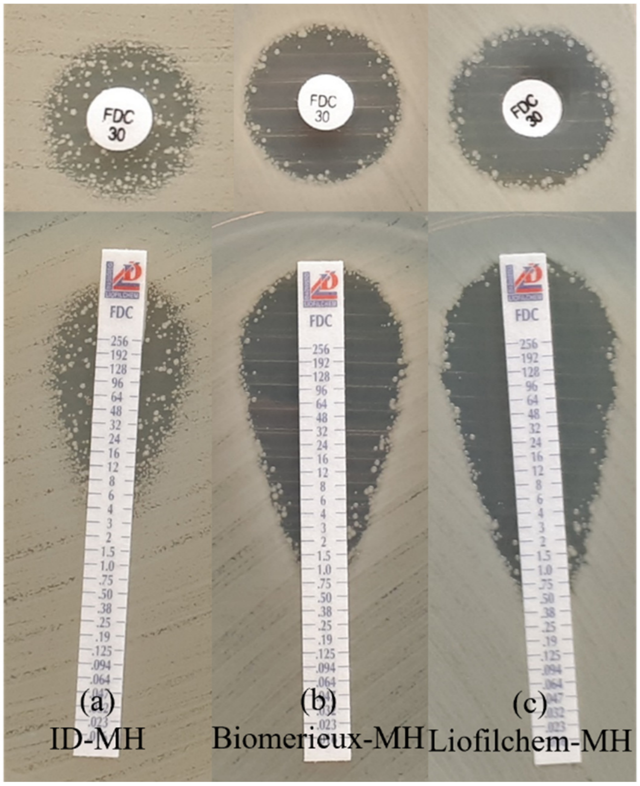

Figure S7. Performance of the DD on ID-MH-agar combined with E-test on ID-MH-agar, UMIC or ComASP. MICs as determined by E-test on ID-MH-agar, UMIC or ComASP (on the x axis) versus DD performed on ID-MH-agar (on the y axis). Colours reflect the MICs determined with the standard BMD method. Categorization errors between the DD/E-test, DD/UMIC and DD/ComASP values (based on the rule whereby by discrepant category resistance overtakes susceptibility) and standard BMD MICs are indicated on top of the count numbers. The red dashed and continous lines denote the CLSI CBPs. The black dashed line denote the regression line. CA, categorical agreement; mE, minor error; ME, major error; vME, very major error.

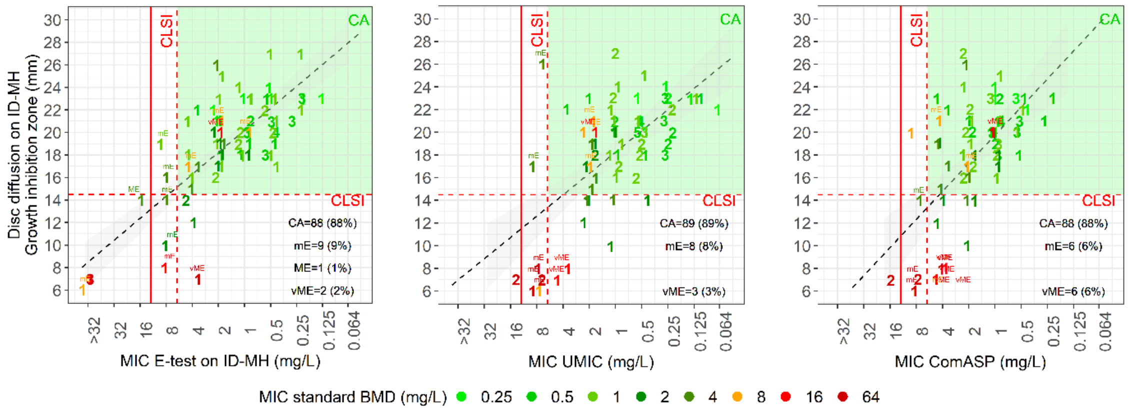

References

1. EUCAST. 2022. European Committee on Antimicrobial Susceptibility Testing. Breakpoint tables for interpretation of MICs and zone diameters Version 120:https://www.eucast.org/fileadmin/src/media/PDFs/EUCAST_files/Breakpoint_tables/v_11.0_Breakpoint_Tables.pdf. (accessed on 14 March 2023).

2. CLSI. 2022. Clinical and Laboratory Standards Institute. Performance Standards for Antimicrobial Susceptibility Testing, 32nd Edition. (accessed on 14 March 2023).
